# Combined frameless stereotactical biopsy and intraoperative cerebral angiography by 3D-rotational fluoroscopy with intravenous contrast administration: a feasibility study

**DOI:** 10.1186/s12880-021-00622-3

**Published:** 2021-06-03

**Authors:** Thomas Linsenmann, Andrea Cattaneo, Alexander März, Judith Weiland, Christian Stetter, Robert Nickl, Thomas Westermaier

**Affiliations:** 1 Department of Neurosurgery, Julius Maximilians University, Josef-Schneider Str. 11, 97080 Würzburg, Germany; 2 Department of Neuroradiology, Julius Maximilians University, Josef-Schneider Str. 11, 97080 Würzburg, Germany

**Keywords:** 3 D rotational fluoroscopy, Neurosurgery, Stereotaxy, Frameless systems, Intraoperative imaging

## Abstract

**Background:**

Mobile 3-dimensional fluoroscopes are an integral part of modern neurosurgical operating theatres and can also be used in combination with free available image post processing to depict cerebral vessels. In preparation of stereotactic surgery, preoperative Computed Tomography (CT) may be required for image fusion. Contrast CT may be of further advantage for image fusion as it regards the vessel anatomy in trajectory planning. Time-consuming in-hospital transports are necessary for this purpose. Mobile 3D-fluoroscopes may be used to generate a CT equal preoperative data set without an in-hospital transport. This study was performed to determine the feasibility and image quality of intraoperative 3-dimensional fluoroscopy with intravenous contrast administration in combination with stereotactical procedures.

**Methods:**

6 patients were included in this feasibility study. After fixation in a radiolucent Mayfield clamp a rotational fluoroscopy scan was performed with 50 mL iodine contrast agent. The image data sets were merged with the existing MRI images at a planning station and visually evaluated by two observer. The operation times were compared between the frame-based and frameless systems (“skin-to-skin” and “OR entry to exit”).

**Results:**

The procedure proves to be safe. The entire procedure from fluoroscope positioning to the transfer to the planning station took 5–6 min with an image acquisition time of 24 s. In 5 of 6 cases, the fused imaging was able to reproduce the vascular anatomy accurately and in good quality. Both time end-points were significantly shorter compared to frame-based interventions.

**Conclusion:**

The images could easily be transferred to the planning and navigation system and were successfully merged with the MRI data set. The procedure can be completely integrated into the surgical workflow. Preoperative CT imaging or transport under anaesthesia may even be replaced by this technique in the future. Furthermore, hemorrhages can be successfully visualized intraoperatively and might prevent time delays in emergencies.

## Background

The aim of stereotactic surgery is to reach a defined target area within a lesion without injuring vessels or eloquent areas by a minimally invasive approach. Therefore, exact preoperative planning of the trajectory is mandatory [1]. Today, functional stereotactic surgery as well as stereotactic tumor biopsy and radio-seed implantation are based on high-quality preoperative MRI. In many centers, the preoperative MRI imaging is fused with a contrast-enhanced CT imaging, which depicts bony structures, soft tissue and the vascular anatomy [1]. Classic ring-based systems have to be fixed to the patient’s head under general or local anaesthesia. The patient has to be transferred to the CT scanner which means for a prolonged period under anesthesia if it is performed under general anesthesia (GA) or increased stress in case of local anesthesia. In addition to the prolonged anaesthetic time, this also means an increased risk of complications [3]. In-hospital transports are associated with significantly increased risks for patients to suffer pulmonary and cardiovascular complications [4].

In addition to frame-based systems, frameless stereotactical systems are increasingly being used [5]. In contrast to frame-based methods, patients do not need CT diagnostics after fixing the ring device under general anaesthesia. The required CT scan with contrast agent can be performed the day before surgery. For this purpose, an additional day of inpatient stay must be considered. In the case of a neurological deterioration under or after the surgical procedure, CT scans are required to evaluate a postoperative hemorrhage. This means another in-hospital transport and time delay.

Recent studies have shown that cerebral aneurysms and hemmorrhages can be successfully visualized intraoperatively with 3-D fluoroscopy [6]. Three-dimensional (3-D) fluoroscopy is an imaging tool that is fast and easily applied. This study evaluated the modalities of image acquisition and image quality of 3-D fluoroscopy after intravenous contrast administration in patients with intracranial tumors undergoing stereotactical procedures.

## Methods

All patients in this analysis were informed of the potential risk of the administration of an iodine contrast agent and radiation exposure, and all patients gave written informed consent.

### Inclusion and exclusion criteria

Patients met the inclusion criteria if they were 18 years of age, had an intracranial tumor with indication for a stereotactical biopsy, and gave written informed consent. Patients were excluded if they had a history of allergy against iodine contrast agent, renal insufficiency, or serum creatinine values (1.2 mg/100 mL).

### Patient positioning and preparation

**Fig. 1:**
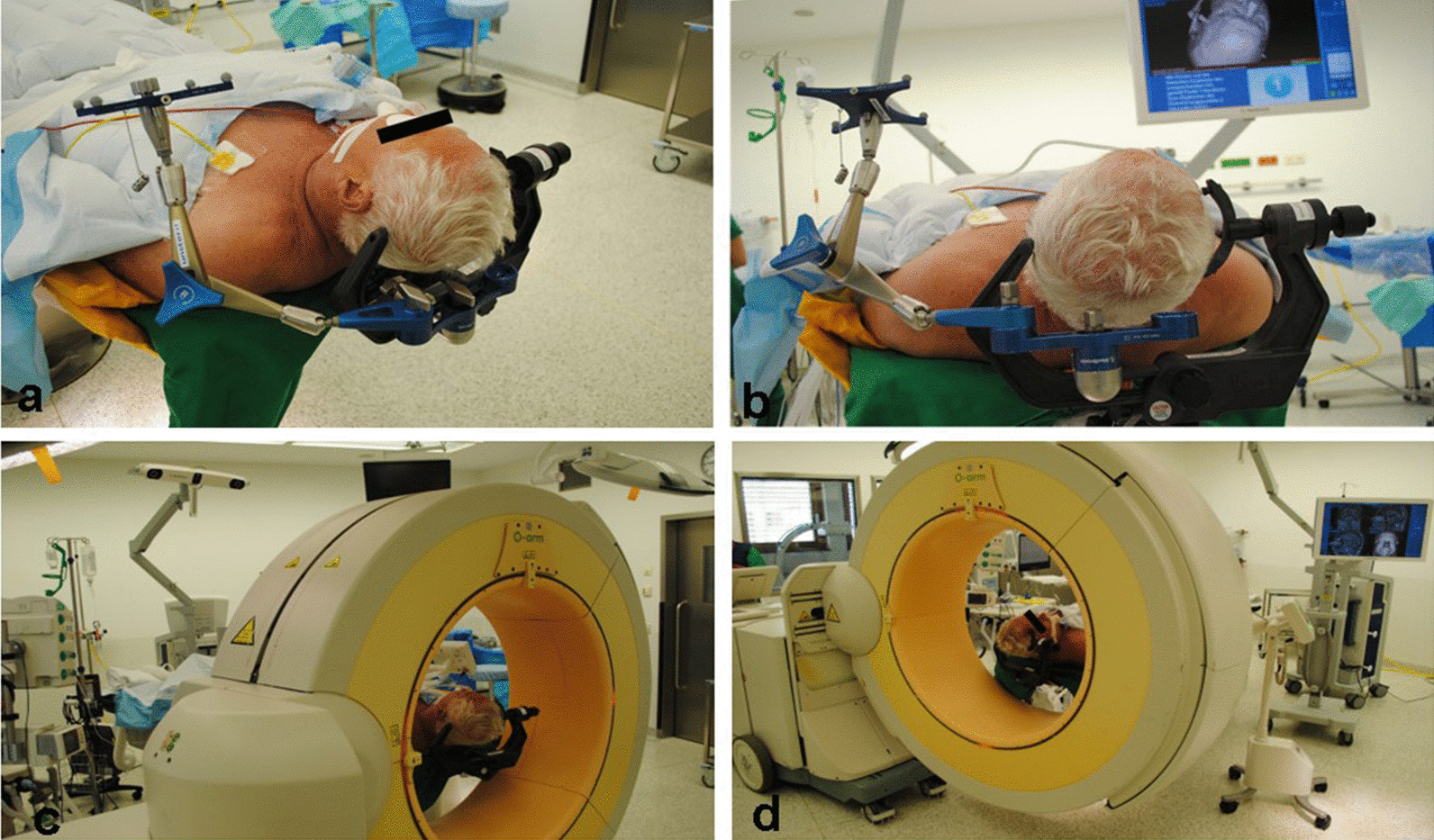
**a**, **b** Patient positioning in a supine position. Head fixation in a radiolucent Mayfield clamp. **c**, **d** The 3-D fluoroscope (O-Arm, Medtronic GmbH, Meerbusch, Germany) position during the hair shaving and surgical disinfection

**Fig. 2:**
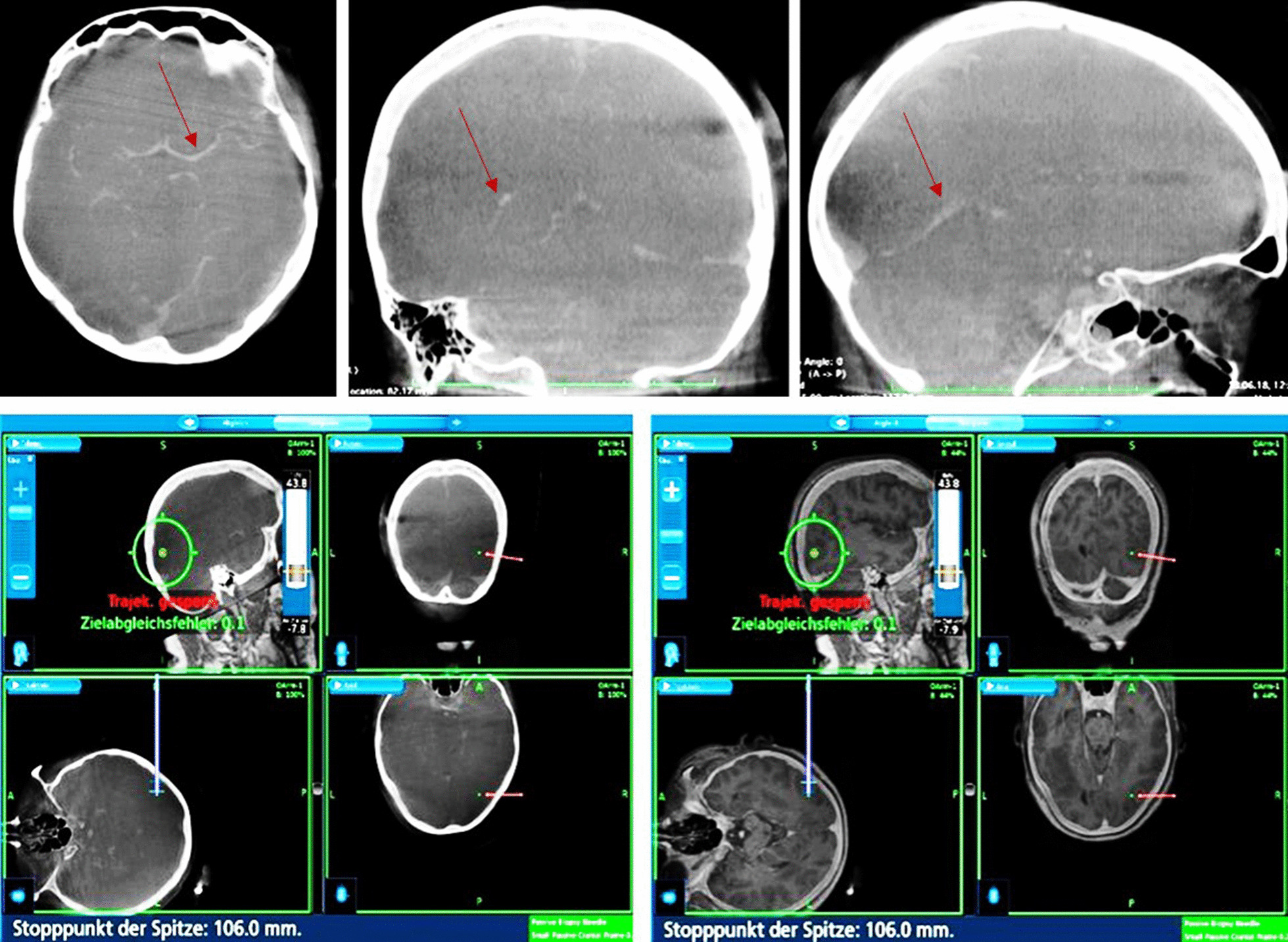
**a**–**c** Acquired images after 3-D rotational fluoroscopy scan with a delay of 12 s after the beginning of the contrast infusion. Visibility of the bony structures and vessel anatomy indicated by red arrows. **d**, **e** DICOM data sets transferation to the MedTronic Stealth Station S7. Merging with standard MRI-MPRage data set. Example for “bone—overlay” (**d**) and 50% MRI—overlay (**e**)

**Fig. 3:**
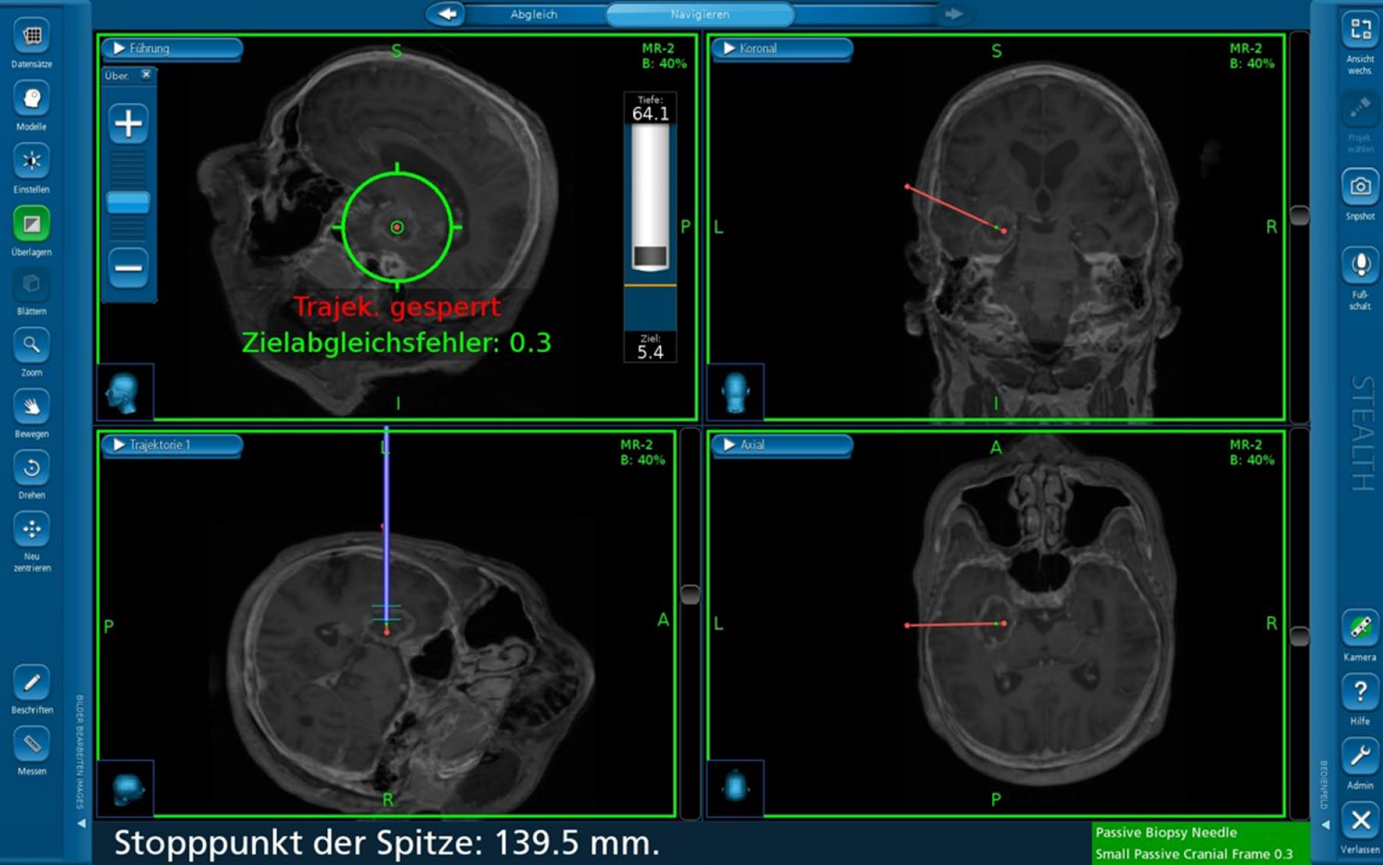
Trajectory planning and stereotactic biopsy under 50% overlay of the MRI image data set

For this study, we adopted the proven setting from our previous projects and adapted it according to the use of a frameless stereotaxic system [6, 7]. After induction of general anesthesia, patients received an arterial line for arterial pressure control in the left radial artery and a central venous line for fluid infusion and contrast agent administration in the right jugular vein. Patients were positioned in a supine position and a radiolucent Mayfield clamp was applied to the head (Integra LifeSciences Corporation, Cincinnati, USA). (Fig. 1a, b) During the hair shaving and surgical disinfection, the 3-D fluoroscope (O-armTM Imaging system (Medtronic, Littleton MA) was placed. (Fig. 1c, d) Acceptable positioning by anteroposterior and lateral fluoroscopy was confirmed. The iodine contrast agent (50 mL Imeron 350, Bracco Imaging, Konstanz, Germany) was then injected manually through a 16-gauge central venous line catheter for 25 s. With a delay of 12 s following the start of the contrast infusion, a 3-D rotational fluoroscopy scan was performed. Seven hundred and fifty images were produced over a span of 24 s (high-definition mode; gantry tilt, 0°; gantry rotation, 360°; image acquisition time, 24 s; reconstruction time, 24 s; standard O-Arm collimator thickness without additional collimation; digital flat panel detector, 40 · 30 cm; camera resolution, 2000 · 1500 (3 megapixels); pixel pitch, 0.194 mm; reconstruction matrix, 512 × 512 × 192). (Fig. 2a–c) Systolic blood pressure was maintained between 100 and 130 mm Hg during contrast injection. DICOM (digital imaging and communications in medicine) data sets were transferred to the StealthStation™ neuronavigation system S7 (Medtronic, Louisville, CO). The data were merged with the standard MRI-MPRage data set (Fig. 2d, e). The quality of the fusion was verified based on the images’ overlay. Known anatomical landmarks and vascular courses served as reference points. In the next step, the trajectory was planned and the navigation devices were fixed at the radiolucent Mayfield clamp followed by anatomical verification of standard landmarks with the navigation pointer. A stereotactically guided skin incision was performed followed by a burr hole trepanation of 9–12 mm in diameter. Serial biopsies were obtained using des Medtronic frameless guided biopsy cannula (Fig. 3). In addition the duration of the operation were compared between the frame-based and frameless systems (endpoint “skin-to-skin”).

### Evaluation of image quality

Two of the coauthors, blinded to each other’s grading, reviewed the reconstructed and merged images and judged the visibility of the bony anatomical landmarks and vessels using the following 4-grade scale: −, no vessels/bone structures visible; (+), poor visibility; +, visibility of the basal vessels/bone structures; ++ good visibility of basal vessels and bone structures including the superior sagittal sinus. An interrater reliability analysis was performed using Kappa statistics in order to determine consistency among raters /reviewers (IBM SPSS Statistics 26, SPSS Worldwide, Chicago, IL, USA).

### Evaluation of time spent

For this purpose, intraoperative protocols were evaluated. The time from “skin to skin” as well as the time span from arrival of the patient in the operating room to leaving (“entry and exit”) were evaluated. The latter parameter also includes the patient's transport to the computed tomography diagnostics in case of frame-based operations, which were evaluated on the basis of 6 representative interventions.

## Results

### Patient characteristics

Six patients were included in this study. Neuropathological findings revealed tumors in four cases (3 Glioblastoma multiforme, 1 Toxoplasmosis). In Patient No. 4 only residuals of an intracerebral hemorrhage were found. In Patient No. 5 neither a tumor nor a infection could be detected (Table 1).

**Table 1:** Patient characteristics

Patient	Age—range	Tumor-localisation	Entity	Merge bones	Merge vessels	Vessels	Vessels dist	Visible tumor	Side effects
1	70–80	Temporal left	GBM	+	+	+	−	−	None
2	50–60	Temporal right	Toxoplamoses	++	++	++	+	−	None
3	70–80	Fronto-parietal right	GBM	++	++	++	+	+	None
4	60–70	Temporal right	Not defined	++	++	+	−	−	None
5	50–60	Temporal right	Not defined	++	++	++	++	−	None
6	60–70	Temporal left	GBM	++	+	++	+	+	None

GBM, Glioblastoma multiforme Grade IV;  − , no vessels/bone structures visible; (+), poor visibility; +, visibility of the basal vessels/bone structures; ++ , good visibility of basal vessels and bone structures including Sinus sagittalis superior

Images 4 a and b have been taken from a male patient who was transferred to the operating room for evacuation of a large right hemispheric intracerebral hematoma. On arrival in the OR, he rapidly developed anisocoria. The surgical team decided to attempt a follow-up imaging using 3D fluoroscopy and intravenous contrast agent in order to verify a possible growth of the hematoma size and to attempt a radiological evidence of a bleeding source.

### Side effects

No cardiovascular side effects or anaphylactic reactions were noted.

### Workflow

After Mayfield clamp fixation, the Medtronic O-arm® was placed during preoperative planning (e.g., shaving, disinfection). With anteroposterior and lateral fluoroscopy, the suitable location was confirmed. Anteroposterior and lateral image acquisitions and 3-D scans took about 2 min and were performed following surgical disinfection of the patient's skin. After the scanning the O-arm^®^ was moved to the parking position and the 3D data set was imported into the Medtronic StealthViz^®^ software of the Stealth Station S7^®^ navigation system. Merging the DICOM data with the MRI-MPrage data set including the trajectory plans took 3 min. In summary, the entire procedure took 5–6 min.

### Technical parameters and radiation exposure

The technical parameters of the fluoroscopy, including radiation exposure, are described in Table 2. The patients received 3-D scans using a high-definition mode with a higher radiation dose to improve image quality. The radiation doses were in the approximate range of a Computed tomography angiography (CTA) according to our previous studies [6, 7] (Table 2).

**Table 2:** Technical parameters

Patient	Age—range	Tumor-localisation	Entity	Tube voltage, KV	Tube current, mA	CTDI, mGy	DLP, mGycm
1	70–80	Temporal left	GBM	120	476.8	34.33	549.05
2	50–60	Temporal right	Toxoplamoses	120	476.8	34.33	549.05
3	70–80	Fronto-parietal right	GBM	120	476.8	64	549.05
4	60–70	Temporal right	Not defined	120	476.8	34.33	549.05
5	50–60	Temporal right	Not defined	110	596	64.27	1027.89
6	60–70	Temporal left	GBM	100	745	67.35	1077.14

GBM, Glioblastoma multiforme Grade IV; CTDI, computed tomography dose index; DLP, dose-length product; n/a, not assigned

### Image quality

The interrater reliability was found to be Kappa = 0.453 (*p* < 0.001). The quality of the images was rated as good both for the resolution of the bony structures and the vessels. Tumor contours could only be safely identified in two patients Only the vascular courses in patient number 4 could not be traced far to the periphery. The authors see the reason for this as the fact that the manual injection of the contrast agent caused a delay, as this procedure does not ensure continuous pressure during the injection phase.

### Operating times

The analysis of both endpoints in the time analysis of the operations resulted in an average duration for “skin-to-skin” of 35 min (range 25–46) for the frameless procedures and 182 min for the time span “entry-exit” (range 160–220). This is contrasted by the significantly prolonged duration of frameless procedures of an average of 51.5 min (range 39–62) “skin-to-skin” and 247 min “entry–exit” (range 110–392).

In both endpoints, the frameless system proves to be faster with less patient time in the operating room (− 67% time “skin-to-skin”; − 73% time “entry–exit”).

## Discussion

This is the first study of the use of intraoperative contrast-enhanced 3-D fluoroscopy instead of a traditional CT-Scan for frameless stereotactic procedures, to the best of our knowledge. In a restricted number of patients, we report the first experiences with this technique. The examination protocol was developed during this study to generate images that display the cerebral vessels consistently via intravenous contrast administration. We rely on the experience of past studies, which were able to demonstrate that the intraoperative use of 3-D fluoroscopy for the imaging of aneurysm is possible [7]. In the field of functional and stereotactical neurosurgery, exact planning of the trajectory is crucial. By many groups, a contrast-enhanced CT scan fused with a preoperative MRI is considered to be the standard imaging in order to increase the accuracy of the anatomy depicted [2].

For frame-based systems, the patient's head must first be clamped in the ring before CT diagnostics with contrast medium injection can be performed under general anaesthesia. This requires an in-hospital transport, followed by a further delay of the point by fusion of the imaging at the planning station. In-hospital transports under analgosedation and mechanical ventilation have a higher risk of complications and delay the surgical procedure [3, 4]. Frameless systems, as used in this study, have the advantage that the patient does not need CT diagnostics under anaesthesia. The required CT with contrast agent injection can be performed the day before the operation and the images can be fused with the MRI data the day before as well. The time required for a stereotactic biopsy is thus reduced.

**Fig. 4:**
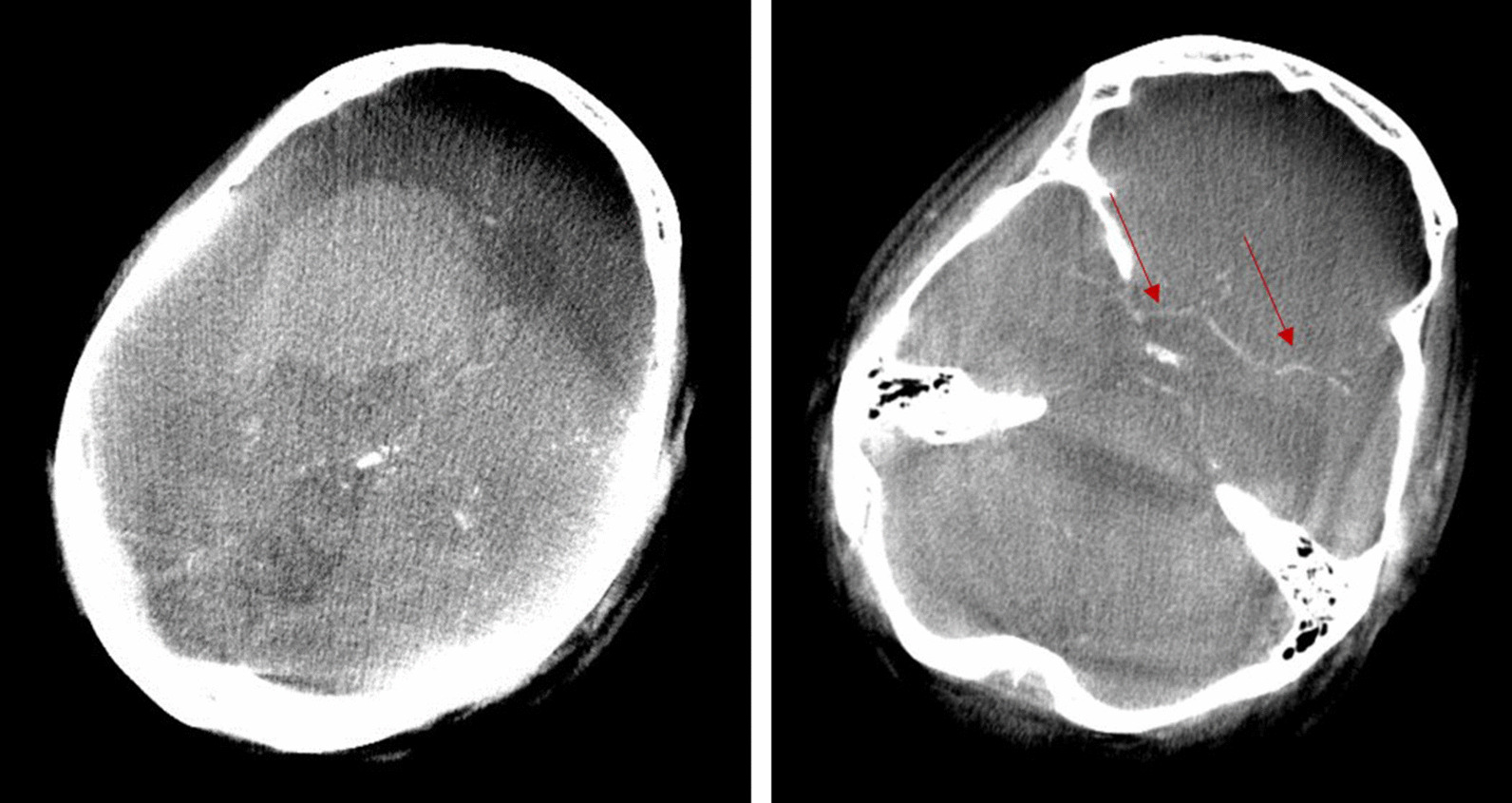
**a**, **b** 3-D fluoroscopy scan detecting a intracerabral hemorrhage. After administration of an iodine contrast agent the vessel anatomy can be well depicted indicated by red arrows

A disadvantage of both procedures is that intraoperative complications, in particular acute bleeding, can only be detected by CT scans after the operation. Thus, there will be a further in-hospital transport causing a delay of therapy. At times, surgeons may notice an ejection of blood through the puncture canal or a hemorrhagic biopsy. This observation, however, is only little sensitive and specific so that some kind of imaging has to be performed. Traditionally, this is done by CT meaning an interruption of surgery for a transport to the CT scanner unless a CT scanner is located in the OR. In case of a hemorrhage, valuable time is lost Intraoperative 3-D fluoroscopy might close this gap in the time line of this acute emergency situation. Figure 4a, b demonstrate that intracerebral hemorrhages can be visualized during the procedure at sufficient quality [2].

The benefits of the 3-D fluoroscopy for intraoperative position control of electrodes and for target and trajectory control have been described [8, 9]. The use of the intraoperative 3-D fluoroscopy is consistently being further developed and applied to other indications and other fields of cranial surgery as well as in the field of (neuro-) radiology to evaluate stent placements and occlusion during endovascular procedures [10].

Fluoroscopy scanners are mobile devices that can be placed wherever and whenever appropriate. Although the O-arm® device is quite large and requires a large operating room, it can be positioned to allow all necessary personnel and equipment to be placed normally [6, 7].

Surgery requires training and practice with the O-arm®. Our surgical team has 10 years of experience with the system, both in complex spinal procedures and in tumor navigation, including pituitary adenomas, in the skull base region. Our assessment is that the acquisition and reconstruction of the images is fast and can be fully integrated into the standard preoperative workflow without any delay if the necessary experience is given.

Since the result of the time analysis is clear and demonstrates a reduced operation time as another advantage of frameless procedures in combination with the intraoperative 3 D rotational fluoroscopy instead the CT scan.

### Limitations

We demonstrate a potential new application field with this project. This study is based on our preliminary work and is intended to give an outlook on the potential this technology may develop. Of course we will conduct the follow-up studies with a correspondingly larger number of observers and patients.

**Fig. 5:**
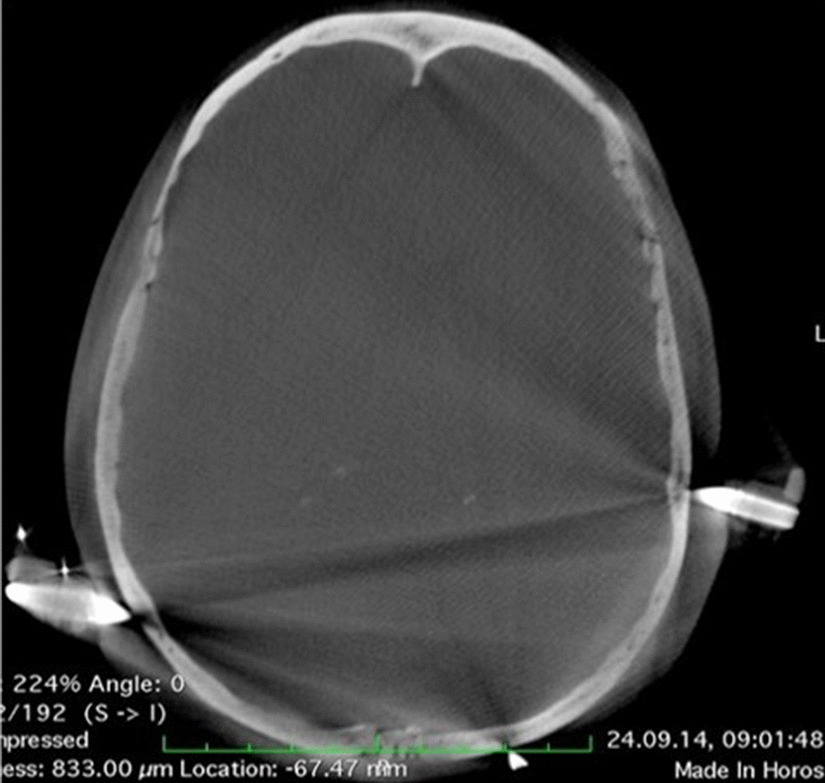
3-D fluoroscopy is susceptible to metal artifacts. Example for susceptible artifacts by using not radiolucent Mayfield pins

**Fig. 6:**
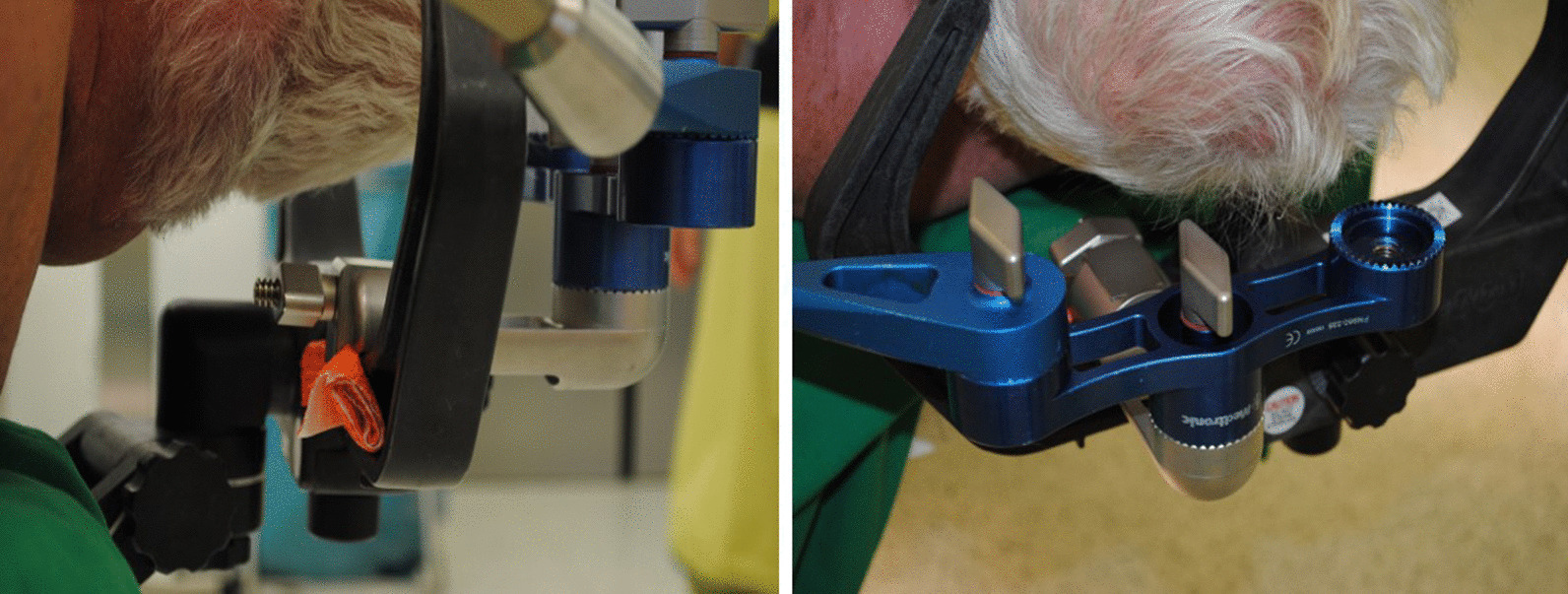
**a**, **b** Intraoperative setting of installed navigation devices with “metal-to-carbon conflict”. Improvised attachment of the metal brackets to the Carbon Mayfield clamp (**a**)

3-D fluoroscopy is susceptible to metal artifacts (Fig. 5). Thus, special radiolucent Mayfield-Pins and a Carbon-Clamp were used. Since the use of equipment from the frameless stereotactic biopsy of the Medtronic Stealth Station® has not yet been combined with intaoperative O-arm® imaging, we developed a modification of the attachment of the guide-arm for the biopsy cannula and the navigation star (Fig. 6a, b). Contrast flow into the cerebral vessels depends on factors such as arterial blood pressure, injection speed, caliber and location of the venous line, and the presence of carotid stenosis. We injected the contrast agent manually via a 14- or 16-gauge central venous line and tolerated systolic arterial blood pressure values between 100 and 130 mm Hg. The factors mentioned above could delay the arrival of contrast and cause inadequate images. The use of an automated injector, tighter arterial blood pressure limits, a slight increase in contrast agent volume, and an algorithm that considers the height of the patient can optimize our present image acquisition protocol.

### Radiation dose

As the system is normally used for spinal neurosurgery, there are no prefabricated profiles available for cranial use. A comparison with other radiological procedures is difficult because the doses used depend very much on the experience of the investigator and the constitution of the patient. The radiation dose of 25 mGy after one image series is similar to doses of native CT or CTA.

### Future prospects

Any department that has experience with 3 d fluoroscopy can easily adopt this technique. The study was conceived to evaluate the effectiveness and feasibility of this technique in combination with a frameless stereotactical system. However, the present series is a pilot study and is too small to reliably validate whether this technique can increase the accuracy of biopsy or positioning of probes and if it may even be able to replace contrast CT for these purposes. The prerequisite for this would certainly be a further improvement and optimization of the radiation dose and contrast-injection protocol. We propose that intraoperative 3-D fluoroscopic angiography could also be a valuable diagnostic tool in emergency cases, e.g. hemorrhages under or following stereotactical procedures. In combination with a target and trajectory verification, which is also proven as a feasible method [8], it may add another useful application field driven by one device.

To reduce artifacts and to avoid damages at the carbon patch-clam, the production of carbon devices instead of metal guiding arms und navigation stars are desirable.

3-D fluoroscopy and image postprocessing are evolving techniques. Therefore, improved discrimination of soft-tissue structures, enhanced vascular and dynamic imaging, and improvements in image processing may be expected in the near future.

## Conclusion

Intraoperative contrast-enhanced 3-D fluoroscopy for stereotactical surgery is feasible and seems to produce images of good quality. It does not interfere with the intraoperative workflow. On the contrary, it may result in a shortening of the gross time of the procedure.

To date, image quality is inferior to CT but even at this stage of development, it can be a useful tool in emergencies.

## Data Availability

The data used to support the findings of this study are available from the corresponding author upon request.

## Abbreviations

CT: Computed tomography
MPRage: Magnetization prepared—rapid gradient echo
MRI: Magnetic resonance tomography
